# Venous Thromboembolism (VTE) Prophylaxis on Discharge Following Major Cancer Surgery in the Abdomen: Improving Compliance With National Guidelines

**DOI:** 10.7759/cureus.73186

**Published:** 2024-11-07

**Authors:** Hassan Iqbal

**Affiliations:** 1 Surgery, Hull Royal Infirmary, Hull, GBR

**Keywords:** colon resection, colorectal cancer, deep vein thrombosis (dvt), general surgery and colorectal surgery, postop complication, postoperative thromboprophylaxis, surgical practice, venous thromboembolism (vte)

## Abstract

Background

Venous thromboembolism (VTE) prophylaxis in hospitalized patients, particularly those undergoing abdominal surgery for cancer, is critical to reducing the incidence of deep vein thrombosis (DVT) and pulmonary embolism (PE). Despite increased awareness, ensuring appropriate VTE prophylaxis post-discharge remains challenging. The National Institute for Health and Care Excellence (NICE) guidelines recommend prolonged prophylaxis in specific cases, yet compliance on discharge often falls short.

Method

A retrospective audit was conducted on 60 patients admitted for elective abdominal cancer surgery in a District General Hospital (DGH) from January to December 2023. This assessed compliance with VTE prophylaxis guidelines, both during admission and post-discharge. Following the first audit cycle, educational interventions for junior doctors, posters in relevant departments, and reminders in electronic patient records were implemented to enhance compliance. A second audit cycle was conducted over four months with 30 patients to evaluate the effectiveness of these interventions.

Results

Initial results showed 100% compliance with VTE assessments and inpatient prophylaxis but only 49.1% compliance with full 28-day post-discharge prophylaxis. Around 24.5% of patients received no further prophylaxis after discharge, while 20.8% exceeded the recommended duration. After the intervention, compliance with recommended VTE prophylaxis improved significantly, with 81.25% of patients completing the prescribed course and only 14.8% receiving inpatient-only prophylaxis. The number of patients exceeding 28 days of prophylaxis decreased from 20.8% to 3.7%. Overall, non-compliance fell from 50.9% to 18.5%.

Conclusion

Simple, targeted interventions, including education and reminders within electronic records, led to significant improvements in VTE prophylaxis compliance post-abdominal cancer surgery. Continued adherence to these strategies, alongside system-embedded reminders, is expected to sustain these improvements and further reduce VTE-related morbidity and mortality.

## Introduction

The awareness of venous thromboembolism (VTE) prophylaxis for inpatients has significantly increased in recent years, largely due to reports of over 14,000 deaths annually from VTE in the UK, marking it as the leading cause of preventable deaths in hospitals [1,2]. While VTE-related deaths have declined, ensuring patients receive continued prophylaxis post-discharge remains challenging. According to the National Institute for Health and Care Excellence (NICE) guidelines, patients undergoing abdominal cancer surgery should continue VTE prophylaxis beyond the hospital stay, if necessary, yet compliance with these recommendations has proven difficult.

Venous thrombosis occurs when a blood clot (thrombus) forms in a vein, leading to restricted blood flow, swelling, or pain. Deep vein thrombosis (DVT) typically develops in the deep veins of the legs, thighs, or pelvis. When part or all of a clot breaks free and travels through the venous system, it can cause a pulmonary embolism (PE). Venous thromboembolism (VTE) encompasses both DVT and PE, affecting approximately one in 1,000 people annually in the UK [3]. The risk of VTE increases with cancer and major surgery, with over half of all cases following hospitalization [4]. Historically, VTE was a major cause of preventable death in hospitals, but improved prevention strategies have significantly reduced associated morbidity and mortality.

General surgical patients are at an elevated risk for VTE, with data suggesting that without proper prophylaxis, 16.8% of these patients will experience a venous thromboembolic event, and 0.9% will die. Administering low molecular weight heparin (LMWH) for VTE prophylaxis reduces the risk of DVT by 72% [5].

## Materials and methods

**Figure 1:**
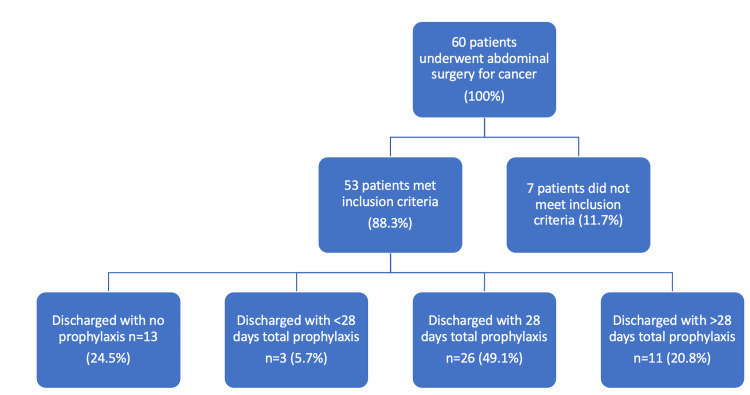
Flow chart of patient inclusion and exclusion based on study criteria and prophylaxis administration, in the first cycle.

The study was conducted at Diana, Princess of Wales Hospital Grimsby in the UK. A baseline audit was conducted on 60 patients admitted between January and December 2023 for elective abdominal cancer surgery. Figure 1 summarizes patients included in the first cycle. Using electronic notes and prescribing data, the audit assessed whether patients were evaluated for VTE risk, received prophylaxis upon discharge, and whether the duration of their prophylaxis adhered to national guidelines. Future VTE diagnoses were also tracked.

Out of the 60 patients, 53 met the criteria for LMWH prophylaxis. The audit found that all patients received VTE assessments and appropriate inpatient prophylaxis, resulting in 100% compliance during the hospital stay. However, only 26 (49.1%) completed the full 28-day course of VTE prophylaxis as recommended. Thirteen patients (24.5%) were discharged without any further prophylaxis, while 11 (20.8%) were prescribed an LMWH course that exceeded the recommended 28-day duration.

These findings indicated that while inpatient prophylaxis adhered to guidelines, there were significant lapses in ensuring patients completed the full course post-discharge. Some patients were discharged on a full 28-day course without accounting for the prophylaxis they had already received during their hospital stay, resulting in over-treatment for 11 patients (20.8%). As these incidents were largely isolated to one period with one set of junior doctors present, it is thought that inappropriate information regarding guidelines was given.

Overall, our results showed that inpatient patients were receiving appropriate prophylaxis prescriptions. However, there was a problem with prescribing VTE prophylaxis and ensuring patients received the correct duration of prophylaxis on discharge.

In this District General Hospital (DGH), it is largely the responsibility of junior doctors to complete VTE assessments and prescribe prophylaxis on admission as well as medication for patients to be discharged.

Junior doctors working in general surgery in this DGH rotate every few months, meaning that they may have been unaware of the policies and importance of accurate VTE prescribing and the need for prolonged VTE prophylaxis for patients with cancer who have undergone abdominal surgery.

A variety of simple education and reminder strategies were implemented. A teaching session was conducted for the new set of junior doctors rotating through the department. Posters were displayed in the general surgery office where daily handovers occur, and consultants were informed of the audit results and asked to remind junior doctors to complete VTE assessments. Additionally, nursing staff were instructed to check whether patients had received appropriate VTE prophylaxis upon discharge.

Furthermore, as a more visible strategy, the project team ensured that appropriate patients had reminders documented on their electronic patient records for doctors to complete a VTE assessment and, where indicated, to suggest prolonged prophylaxis. This was aimed at reducing errors and serving as a final reminder for the discharging doctor to consider VTE prophylaxis when discharging patients.

## Results

**Figure 2:**
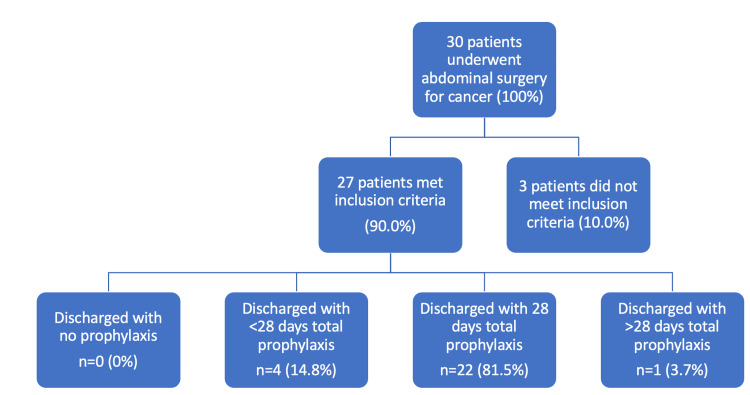
Flow chart of patient inclusion and exclusion based on study criteria and prophylaxis administration, in the second cycle.

In the second audit cycle, after the implementation of educational and procedural interventions, overall compliance with VTE prophylaxis guidelines improved substantially. The reassessment was conducted over a four-month period, involving 30 patients who underwent elective abdominal surgery for cancer. Out of these, 27 met the inclusion criteria, and the results indicated significant progress in terms of adhering to national guidelines, as shown in Figure 2.

One of the most notable improvements was in the proportion of patients who were fully compliant with VTE prophylaxis guidelines. In the first audit cycle, only 49.1% of patients completed the full 28-day prophylactic course or received appropriate guidance for continuation post-discharge. However, after implementing the educational interventions, this percentage increased to 81.25%. This suggests that the reminder system and educational efforts were effective in ensuring that the majority of patients received proper VTE prophylaxis according to national guidelines.

There was also a notable decrease in the proportion of patients who received VTE prophylaxis only during their inpatient stay without being prescribed the appropriate continuation post-discharge. Initially, 30.2% of patients fell into this category. Following the interventions, this percentage nearly halved, dropping to 14.8%. This demonstrates a marked improvement in ensuring that VTE prophylaxis was continued appropriately after discharge, reducing the risk of thromboembolic events in the outpatient setting.

Another issue identified in the baseline measurement was that 20.8% of patients were prescribed a 28-day course of VTE prophylaxis at discharge, which did not account for the days of LMWH prophylaxis already received during their inpatient stay. This led to some patients exceeding the recommended duration of 28 days. In the second cycle, the percentage of patients receiving VTE prophylaxis beyond the recommended 28 days dropped to 3.7%, indicating a sharp improvement. This reduction highlights the success of the intervention in ensuring that discharge prescriptions were more accurately tailored to the patient’s overall course of prophylaxis.

**Figure 3:**
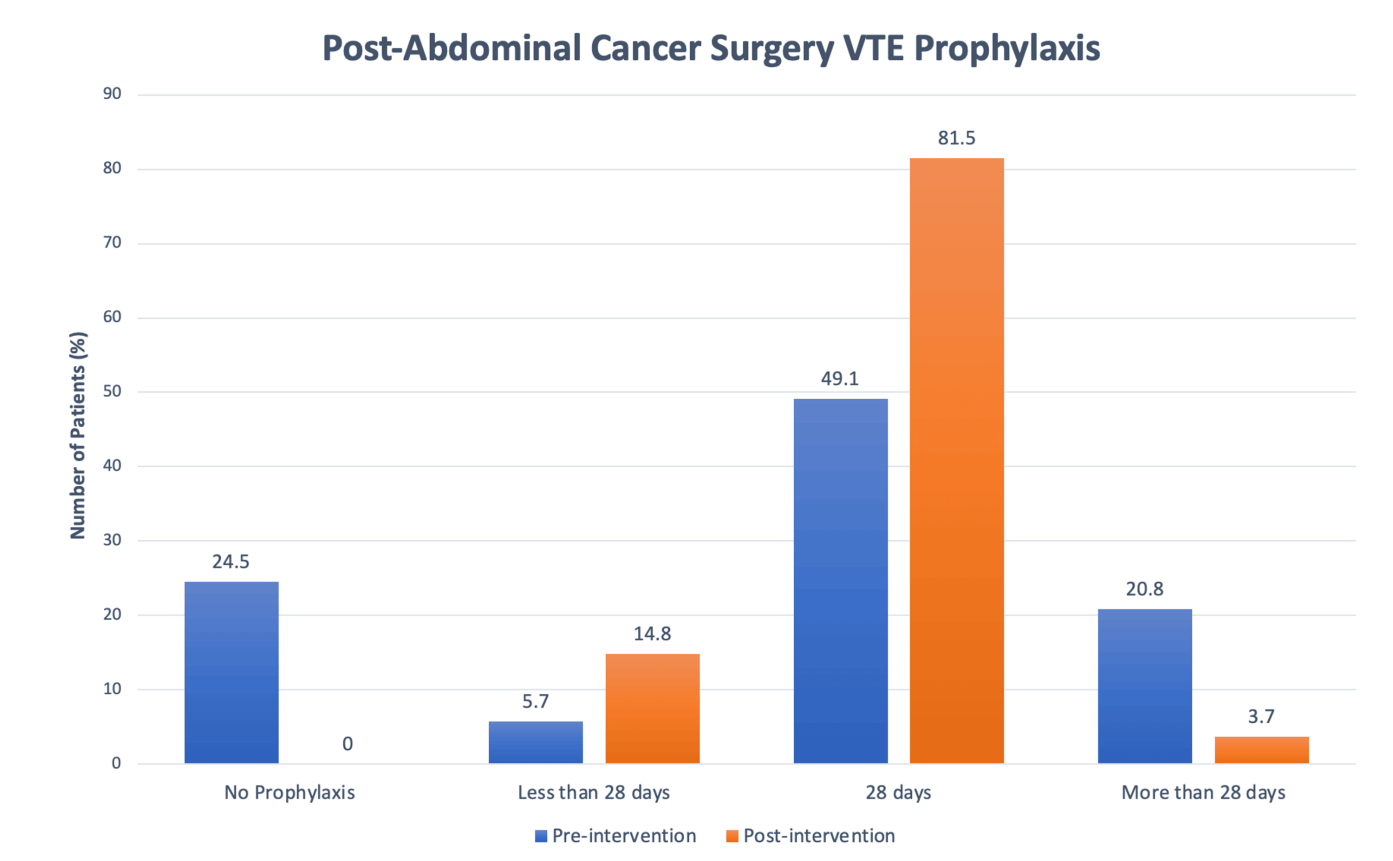
VTE prophylaxis post-abdominal cancer surgery, comparing cycle 1 and cycle 2. VTE: venous thromboembolism

The total number of patients receiving incorrect prophylaxis (whether insufficient or excessive) decreased, as shown in Figure 3. In the first cycle, 50.9% of patients did not receive the appropriate duration of VTE prophylaxis, either receiving no continuation of prophylaxis upon discharge or being prescribed more than 28 days unnecessarily. Following the interventions, this figure dropped to 18.5%. This shows that the combined measures of education, reminders, and systemic improvements led to a major reduction in the overall errors associated with prescribing VTE prophylaxis.

## Discussion

This quality improvement project underscores how targeted, simple interventions can yield significant improvements in clinical practice, particularly in adherence to national guidelines for VTE prophylaxis in patients undergoing abdominal surgery for cancer.

The interventions implemented, such as educational sessions for junior doctors, visual reminders like posters, and system-based prompts, proved highly effective in improving compliance. This success aligns with evidence from other studies that emphasize the importance of education, reminders, and guideline reinforcement in improving clinical practices [6,7]. For example, interventions such as regular education and electronic alerts have been shown to improve compliance with best practices in various healthcare settings [8]. The findings reinforce that embedding guideline reminders into routine processes can ensure that they are considered during crucial moments, such as patient discharge.

One of the key reflections from this project is the importance of continuous education, especially in environments with frequent staff rotations, as in this District General Hospital (DGH). Junior doctors, who are primarily responsible for prescribing discharge medications, often rotate every few months, which can lead to variability in adherence to guidelines based on their knowledge and experience. This was clearly evidenced in the first cycle, where a cohort of doctors discharged patients with inappropriate durations of VTE prophylaxis, likely due to misinformation or a lack of understanding of the national guidelines.

This finding is consistent with broader literature, which highlights that lack of education or misinterpretation of guidelines is a common issue in preventing guideline adherence [9,10]. However, targeted educational sessions, as implemented in this project, are known to have a positive impact on improving staff adherence to protocols [11-13]. The project showed that providing junior doctors with clear, concise information at the start of their rotation significantly improved compliance rates.

One limitation of this project is the potential loss of knowledge after junior doctors rotate out of the department. While the initial educational interventions were effective, this approach relies on consistent re-education of new staff, which may not always be sustainable in the long term [14,15]. This is a common challenge in healthcare, particularly in settings with high staff turnover. Without a continuous education program or a more permanent solution, compliance could regress when new cohorts of junior doctors join the department [16,17].

To address this, it might be beneficial to formalize the educational sessions as part of the hospital’s orientation program for all new surgical staff, ensuring the knowledge is passed on to every new group of junior doctors [15]. Furthermore, embedding guideline prompts directly into electronic systems, as done in this project, is a long-term, sustainable strategy for maintaining compliance [8,18]. Literature suggests that computerized decision support systems are highly effective in ensuring adherence to best practices, as they provide real-time reminders and minimize the risk of human error [19,20].

While the integration of VTE guideline prompts into the discharge software was a useful intervention, it raises the potential issue of over-reliance on electronic systems [20-22]. Healthcare professionals may become dependent on automated prompts, potentially reducing their initiative to actively engage with and understand the guidelines [20]. This phenomenon, sometimes referred to as "alert fatigue," occurs when clinicians are bombarded with electronic prompts, leading them to overlook or ignore important alerts [23]. To mitigate this, it is crucial to strike a balance between automated reminders and the cultivation of clinical knowledge through continuous education.

With 60 patients in the first cycle and 30 in the second, the findings are somewhat limited in their generalizability. While the improvements observed were significant, a larger sample size would provide more robust evidence of the intervention's effectiveness. Additionally, the project was conducted in a single DGH, which may limit its applicability to other hospital settings. Factors such as hospital size, staffing levels, and the availability of resources could influence the success of similar interventions elsewhere.

Further research involving larger patient cohorts and multiple hospital settings would be valuable in validating these findings and assessing whether similar strategies could be implemented more broadly. In addition, longer follow-up periods may provide insight into whether the improvements in compliance with VTE prophylaxis guidelines are sustained over time.

An additional reflection relates to the importance of a multidisciplinary approach in ensuring guideline compliance. While junior doctors are typically responsible for discharge prescriptions, nurses and senior doctors play an essential role in overseeing patient care and ensuring that national guidelines are followed. In this project, nursing staff were involved in checking whether patients had received appropriate VTE prophylaxis before discharge, which likely contributed to the improvements seen in the second cycle.

This collaborative, team-based approach has been shown to be effective in improving adherence to clinical protocols [24]. Studies have demonstrated that when multiple members of the healthcare team are involved in patient care decisions, there is a greater likelihood of compliance with best practices. Ensuring that all team members are aware of and engaged with VTE prophylaxis guidelines can help maintain high standards of care even as junior doctors rotate [24,25].

## Conclusions

Following these basic and simple interventions, there has been considerable improvement in meeting national guidelines for VTE prophylaxis following major abdominal surgery for cancer. It is hoped that improvements made to the computer systems to remind doctors of VTE prophylaxis for qualifying patients will maintain these improvements and ultimately lead to a further decline in venous thromboembolic events following surgery in this DGH.
